# The Rush Caregiver Initiative: A Model for Caregiver Health and Wellness in Age-Friendly Health Systems

**DOI:** 10.1093/geroni/igab046.1189

**Published:** 2021-12-17

**Authors:** Leslie Pelton, Ellen Carbonell, Robyn Golden

**Affiliations:** 1 Institute for Healthcare Improvement, Boston, Massachusetts, United States; 2 Rush University Medical Center, Chicago, Illinois, United States

## Abstract

The Rush Caregiver Health and Well-Being Initiative (Caregiver Initiative) draws together evidence-based practices into a single framework to improve care for older adults and caregivers. The Caregiver Initiative has two components: system-level and caregiver level interventions. The complexities of system change take place within leadership, data management, and provider teams throughout the health care system, and solutions to resistance have been developed. Caregiver-level interventions start with an assessment using evidence-based tools, and offer an opportunity to participate in a Teach-Back Clinic, Family Care Planning sessions, and/or Goals of Medical Care meetings, all connected to the 4Ms of an Age-Friendly Health System. Contact and follow-up issues were addressed, and as of February 2021, 191 caregivers have enrolled. Outcomes to date show statistically and clinically significant reductions in depression, anxiety, and caregiver burden. This presentation will highlight lessons learned in the development of the model and caregiver outcomes to date.

